# Imaging Parkinson’s disease below the neck

**DOI:** 10.1038/s41531-017-0017-1

**Published:** 2017-04-25

**Authors:** Per Borghammer, Karoline Knudsen, Tatyana D. Fedorova, David J. Brooks

**Affiliations:** 1 Department of Nuclear Medicine & PET Centre, Institute of Clinical Medicine, Aarhus University, Aarhus, Denmark; 2 Division of Neuroscience, Department of Medicine, Imperial College London, London, UK; 3 Division of Neuroscience, Newcastle University, Newcastle upon Tyne, UK

## Abstract

Parkinson’s disease is a systemic disorder with widespread and early *α*-synuclein pathology in the autonomic and enteric nervous systems, which is present throughout the gastrointestinal canal prior to diagnosis. Gastrointestinal and genitourinary autonomic symptoms often predate clinical diagnosis by several years. It has been hypothesized that progressive *α*-synuclein aggregation is initiated in hyperbranched, non-myelinated neuron terminals, and may subsequently spread via retrograde axonal transport. This would explain why autonomic nerves are so prone to formation of *α*-synuclein pathology. However, the hypothesis remains unproven and in vivo imaging methods of peripheral organs may be essential to study this important research field. The loss of sympathetic and parasympathetic nerve terminal function in Parkinson’s disease has been demonstrated using radiotracers such as ^123^I-meta-iodobenzylguanidin, ^18^F-dopamine, and ^11^C-donepezil. Other radiotracer and radiological imaging methods have shown highly prevalent dysfunction of pharyngeal and esophageal motility, gastric emptying, colonic transit time, and anorectal function. Here, we summarize the methodology and main findings of radio-isotope and radiological modalities for imaging peripheral pathology in Parkinson’s disease.

## Introduction

Parkinson’s disease (PD) is a systemic disorder with widespread *α*-synuclein pathology in the peripheral and enteric nervous systems.^1^ Extensive pathology is seen in the parasympathetic and sympathetic nervous system and throughout the gastrointestinal canal in diagnosed PD cases and in the prodromal disease phase.^2–6^ Genitourinary and gastrointestinal autonomic symptoms also predate clinical diagnosis.^7–9^ It has been hypothesized that *α*-synuclein aggregation is initiated in hyperbranched, non-myelinated neuron terminals, and subsequently spreads via retrograde axonal transport. This hypothesis would also explain the predisposition of autonomic nerves to form early and severe *α*-synuclein pathology.^10, 11^

Functional imaging tools are important for elucidating the nature and chronology of peripheral pathology in PD. Radio-isotope studies have the ability to directly assess the loss of cellular structures, including parasympathetic and sympathetic nerve endings. Other imaging methods can determine the functional consequences of these pathologies. Dysphagia, gastric emptying, intestinal transit times, and anorectal dysfunction can all be quantitated through the use of radiotracer and radiological imaging techniques. Here, we summarize the methodology and main findings of these diverse methods for imaging the periphery in PD.

## Imaging the sympathetic nervous system

The sympathetic post-ganglionic nerve terminals can be visualized with the norepinephrine analogue ^123^I-meta-iodobenzylguanidin (MIBG). The tracer accumulates and is stored in the vesicles of sympathetic terminals, and is therefore an in vivo marker of sympathetic terminal integrity. Other tracers for imaging sympathetic terminals include the PET ligands ^18^F-fluorodopamine and ^11^C-meta-hydroxyephedrine (HED).^12, 13^

In the majority of PD patients, *α*-synuclein pathology is seen in sympathetic ganglia and the intermediolateral column of the medulla.^2, 14^ It has been shown that *α*-synuclein initially accumulates in distal, cardiac sympathetic axons, followed by proximal accumulation in the neuronal cell bodies.^15^ The density of ganglionic *α*-synuclein inclusions is greater than in the intermediolateral column.^14^ Therefore, it has been suggested that a distal-to-central spreading sequence of *α*-synuclein aggregates occurs in the sympathetic neurons.

### Cardiac imaging

**Fig. 1:**
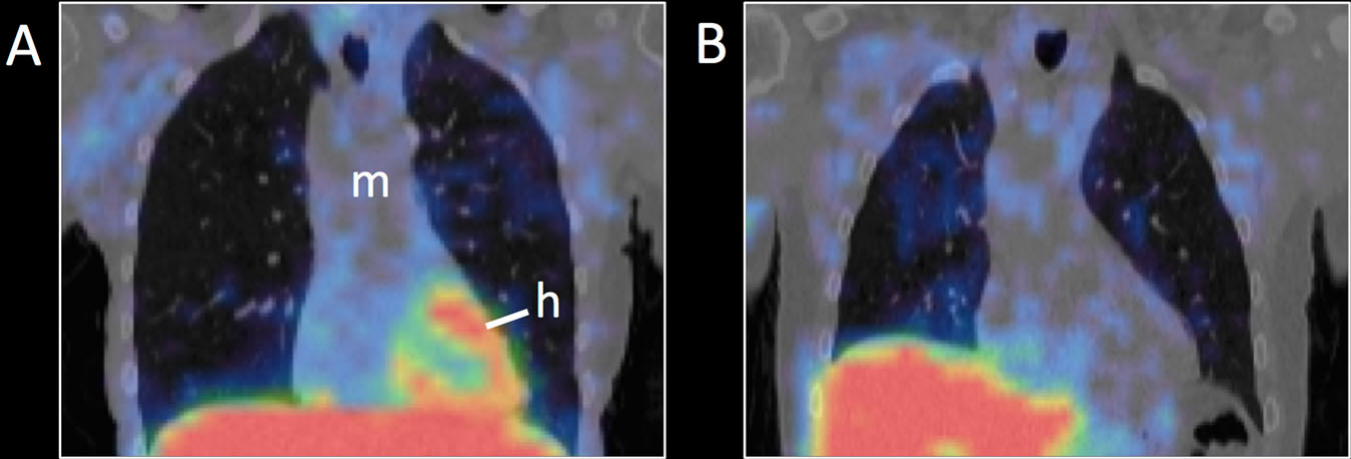
^123^I-MIBG SPECT images superimposed on anatomical CT. **a** Healthy subject with normal cardiac uptake. **b** PD patient with severely decreased cardiac uptake. [h heart, m mediastinum]

Sympathetic cardiac imaging in PD has been the subject of several recent reviews,^16–18^ and will be only briefly summarized here. An early (15 min) and late (3–4 h) MIBG image is acquired using a gamma camera. The images are interpreted by qualitative visual inspection and a heart-to-mediastinum (H/M) ratio is calculated. A nearly total loss of cardiac signal is seen in most PD patients (Fig. 1). The purpose of early and late images is to estimate tracer delivery and vesicular storage.^19^ The majority of healthy subjects showing increasing H/M values from early to later time points, whereas decreasing ratios are seen in most PD and DLB patients.^20^ In later years, several groups have shown that three-dimensional (3D) tomographical MIBG imaging yields superior diagnostic performance, since segmental cardiac denervation can more easily be discerned.^21, 22^

Cardiac MIBG signal is reduced in 80–90% of PD and DLB patients in total, but only in ~ 60% de novo PD patients.^18, 23–26^ Patients with progressive supranuclear palsy, corticobasal degeneration, and multiple system atrophy usually exhibit normal cardiac innervation.^20, 23^ Recent meta-analyses showed overall sensitivity and specificity of 80–85% for differentiating PD from atypical movement disorders.^27, 28^ Interestingly, nearly all RBD patients, now known to be prodromal PD or DLB in the majority of cases,^29^ show decreased cardiac MIBG uptake more similar to later stage PD patients, and clearly surpassing the signal reduction seen in newly diagnosed PD patients without RBD.^30, 31^ A recent ^11^C-HED PET study demonstrated that PD patients with mildly affected baseline scans showed progressive decline preferentially in infero-lateral segments. In contrast, patients with severely denervated infero-lateral wall segments at baseline showed progressive signal loss in the anterior and septal segments, suggesting a pattern of “catch-up” eventually leading to global left ventricle denervation.^13^

Decreased cardiac MIBG uptake is more pronounced in akinetic-rigid PD patients compared to tremor-predominant patients.^32^ However, the relationship between cardiac denervation and disease stage is controversial. Some studies showed correlations between cardiac MIBG signal and Hoehn and Yahr (H&Y) stage and also with progressive Unified Parkinson's Disease Rating Scale motor scores, whereas other studies failed to detect such correlations.^16^

Orthostatic hypotension is common in medicated PD patients,^33^ but it shows limited correlation with cardiac MIBG signal. Especially early PD cases with pathological heart scans often show no signs of orthostatic hypotension.^34–36^ In a fluorodopamine PET study, while all PD patients with orthostatic hypotension showed diffusely decreased myocardial uptake, 50% of those without orthostatic hypotension showed some fluorodopamine retention primarily in the septum,^37^ suggesting a potential relationship between the sympathetic imaging markers and orthostatic hypotension. This may only be discernible when 3D tomographic imaging is employed.

It should be noted that loss of cardiac sympathetic signal is not specific to PD. Diabetic patients with cardiac autonomic neuropathy^38^ and patients with chronic heart failure^39^ exhibit similar reductions in cardiac MIBG signal. Furthermore, a range of medications are known to affect MIBG uptake, including certain antidepressants, phenylephrine, cocaine, labetalol, and other drugs,^40^ and it is recommended that patients abstain from these medications prior to MIBG imaging.

### Imaging other regions

The thyroid gland is clearly discernible on a normal MIBG image (Fig. 1a), and the thyroid is heavily innervated by sympathetic nerves.^41^ Marked reductions in thyroid uptake of ^18^F-fluorodopamine and MIBG have been reported in PD patients^37, 42^ (Fig. 1b). Interestingly, two studies reported similar reductions in cardiac MIBG uptake of PD patients, heart failure patients, and diabetic patients with neuropathy, but only the PD patients displayed a reduction in thyroid uptake.^38, 43^ These findings suggest that thyroid sympathetic denervation may be more specific for PD, and may enhance the specificity of MIBG imaging for correctly diagnosing PD.

No reduction was seen in uptake of MIBG by the liver or lungs in PD.^42^ To our knowledge, it has not be determined whether MIBG liver signal reflects its sympathetic innervation, or rather non-specific liver accumulation of the tracer and its metabolites in the process of tracer clearance. Some MIBG signal is always seen in the pulmonary tissue (Fig. 1), but the observation that reserpine-treated rodents displayed increased MIBG uptake in the lung with concomitant decrease in heart uptake suggests that the pulmonary MIBG uptake is non-specific.^44^

To summarize, moderate-to-late stage PD cases and nearly all DLB patients show reduced cardiac accumulation of MIBG and fluorodopamine, as do most RBD patients. In contrast, only ~ 60% of de novo PD cases show measurable sympathetic denervation, an observation, which underscores that RBD is a unique PD phenotype. Also, the sizeable fraction of early stage PD patients with normal cardiac MIBG signal somewhat limits the clinical utility of this imaging modality. Some investigators reported correlations between motor symptom severity and loss of cardiac MIBG signal, but cardiac sympathetic denervation on imaging displays limited correlation with autonomic symptoms. Reduced MIBG and fluorodopamine uptake is also detectable in the thyroid gland of most PD patients, but the clinical relevance of this observation is uncertain.

## Imaging the parasympathetic nervous system

It has been suggested that PD initiates in the autonomic nerve endings in the gastro-intestinal mucosa, perhaps induced by a hitherto unknown toxin, which sets in motion a cascade of pathological events.^45^ The disease process may then spread through the vagal nerve from the gastrointestinal tract to the central nervous system (CNS).

This hypothesis would explain the observation that the dorsal vagal motor nucleus (DMV) is the primary target structure in the brain stem of most PD patients.^46^ The idea is also supported by the common finding of phosphorylated *α*-synuclein inclusions in the vagus nerve^2^ and the ~ 50% neuron loss in the DMV of PD patients.^47, 48^ In the periphery, *α*-synuclein aggregates are seen in vagal efferents of internal organs,^49–51^ including the intestinal myenteric and submucosal plexus.^52–54^ The severity of *α*-synuclein pathology in the gastro-intestinal tract exhibits a rostro-caudal gradient, with the highest density of Lewy pathology in the upper gut,^2^ a distribution which correlates with vagal innervation of the gut.^55^

These findings support the hypothesis that the parasympathetic nervous system is among the first sites to be affected in PD. The findings also suggest that disease progression from the periphery to central structures may partly follow parasympathetic neuronal pathways, although this hypothesis remains unproven. Of note, two independent epidemiological studies recently showed that total truncal vagotomy reduces the subsequent risk of PD by 40–50% (refs 56, 57), which emphasizes the important role of the parasympathetic nervous system in the understanding of PD pathogenesis. In vivo imaging of parasympathetic integrity in PD could thus provide a unique insight into disease pathophysiology as well as an improved understanding of disease initiation and progression.

Cholinergic in vivo imaging of central brain structures in dementia disorders has been used for many years^58^ and PET and SPECT ligands are available for several molecular targets. Pre-synaptic targets include the vesicular acetylcholine transporter (VAChT) and the breakdown enzyme acetylcholinesterase (AChE). Post-synaptic targets are the muscarinic and nicotinic acetylcholine receptors, which are abundantly expressed in the brain.^58^ However, despite longstanding availability of radiotracers with affinity for cholinergic targets almost no research has been done in the field of parasympathetic imaging—possibly since parasympathetic neurons do not express specific molecular targets to distinguish them from other cholinergic connections.

Historically, studies have assessed the integrity of parasympathetic, cholinergic pathways in peripheral tissue using measurements of AChE activity.^59–61^ This method has now largely been replaced by VAChT immunohistochemistry, which is more specific for cholinergic neurons in general, but is also not specific for parasympathetic fibers. In the gut, more than 50% of enteric neurons are cholinergic.^21^ Parasympathetic imaging of internal organs poses an additional challenge in the form of rapid hepatic metabolism and subsequent biliary excretion of many radiotracers. Thus, radio metabolites in the intestinal lumen may bias imaging results of the gut and neighboring organs. For this reason, radiotracers with slow biliary excretion or exclusively renal excretion are mandatory.

**Fig. 2:**
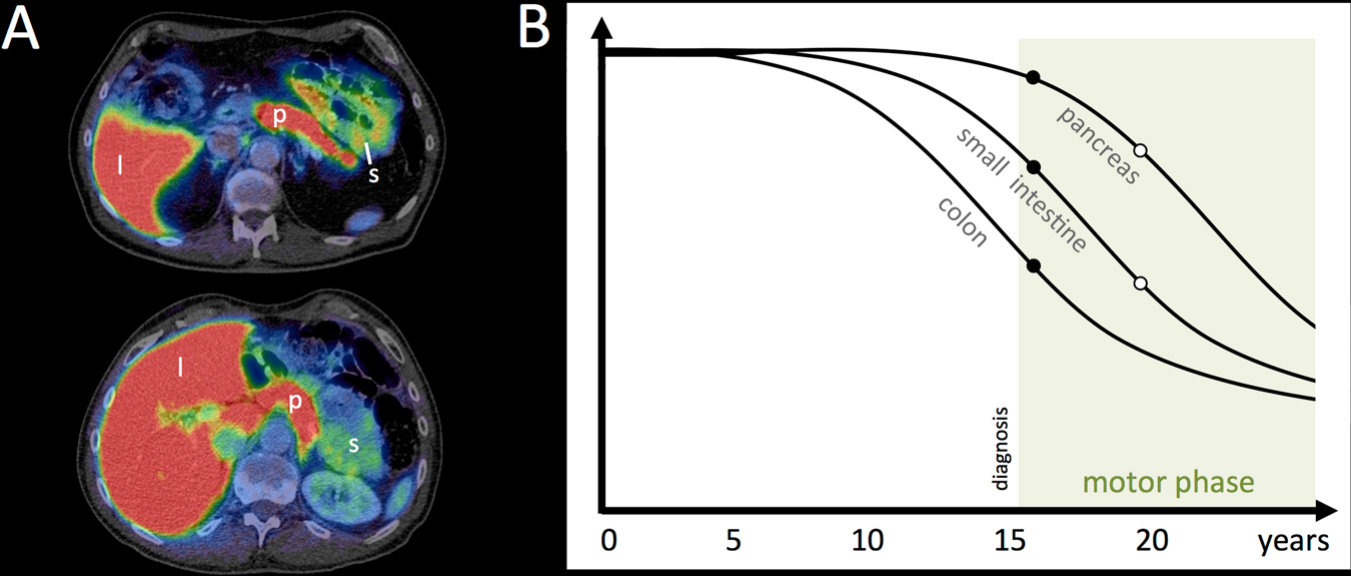
^11^C-donepezil PET/CT images. **a** Summed PET images superimposed on anatomical CT in a healthy control (*top*) and a PD patient (*bottom*). Note the visually apparent decrease in the small intestine signal. **b** Hypothetical timeline of parasympathetic denervation during the course of prodromal and manifest PD. *Closed* and *open circles* represent ^11^C-donepezil PET data from de novo (duration 1 year) and moderate stage PD patients (duration 4.5 years), respectively.^65, 66^ Shortly after diagnosis, some ^11^C-donepezil signal loss is seen in the small intestine and colon, but not in the pancreas. Five years after diagnosis, progressive signal loss is seen in the pancreas, small intestine, and colon. [l liver, p pancreas, s small intestine]

The PET tracer [^11^C] donepezil has been successfully utilized to measure AChE density in the brain of patients with Alzheimer’s disease or PD.^62, 63^ We recently implemented this tracer for quantification of AChE density in peripheral organs of healthy individuals and showed that the distribution of [^11^C] donepezil approximates vagal innervation of the internal organs.^64^ Two [^11^C] donepezil PET studies of 12 early-to-moderate stage PD patients and 19 newly diagnosed patients have been performed. In the early-to-moderate PD group (disease duration 4.5 years) the PET signal was markedly decreased in the small intestine (35%) (Fig. 2a) and pancreas (30%) compared with healthy controls.^65^ In this first study, the colon was not assessed. In contrast, newly diagnosed PD patients (duration ~ 1 year) displayed a less pronounced decrease of 14 % in the small intestine, no decrease in pancreatic signal, but a significant 22% decrease in the colon.^66^ These findings suggest variable rates of progression in different internal organs, with the colon being the most severely affected, followed by the small intestine, and then the pancreas (Fig. 2b). However, this hypothetical progression needs to be demonstrated in longitudinal PET studies of PD patients and ideally also RBD patient cohorts. It should also be noted that although ^11^C-donepezil shows no biliary excretion during a 60 min PET scan, we observed some direct trans-mural tracer excretion across the stomach mucosa, which may bias correct estimates of the intestinal PET signal.^66^ Thus, a future research goal would be the identification of a cholinergic PET tracer complete free from biliary or trans-mural excretion.

## Functional imaging of the gastrointestinal tract

The full spectrum of gastrointestinal symptoms in PD was recently reviewed in detail,^67, 68^ and is only briefly summarized here. The main focus in the present review will be on imaging methods utilized for studying gastrointestinal functional alterations in PD.

### Oro-pharynx and esophagus

The pooled prevalence of subjective dysphagia in PD is 35% (ref. 69) and aspiration is also commonly seen.^70^ Dysphagia usually emerges in late-stage PD, but on occasion can be the presenting feature.^71^

Dysphagia may in part be caused by bradykinesia and rigidity secondary to basal ganglia dysfunction, but the exact underlying pathophysiology of dysphagia remains to be determined. The esophageal motility patterns are mainly determined by nuclei in the medulla oblongata, and vagotomy results in upper esophagus paralysis, which impairs food propulsion.^72^ Theoral part of the esophagus receives vagal innervation from the nucleus ambiguus, whereas the distal esophagus is innervated from the DMV.^73^ Pathological *α*-synuclein inclusions are present in both of these vagal nuclei, although much more in the DMV.^46, 74^ Recently, it was shown that pathology in peripheral motor and sensory pharyngeal nerves may also contribute to oropharyngeal dysphagia.^75^

Dysphagia can be examined by fluoroscopic barium studies, where patients ingest a thin barium liquid or barium-coated bread during a continuous X-ray.^76^ Oro-pharyngeal transit time and swallowing efficiency can then be determined.

In a comprehensive video fluoroscopy study of 72 PD patients, epiglottic dysmotility was detected in 56% of PD patients, pharynx constrictor dysfunction in 42%, esophagus dysmotility in 91%, and reflux in 56% (ref. 77). A similar study reported disturbances of the oro-pharyngeal phase in 75% of PD patients.^78^ Silent aspiration is also frequently seen in PD patients. More recently, a study failed to detect perturbed swallowing function in PD patients with and without dyskinesias compared to normal controls. Perhaps surprisingly, the swallowing efficacy was somewhat decreased in patients without dyskinesia compared to the dyskinetic patients, which could be caused by higher L-dopa intake in the latter group.^79^

Dysphagia can also be studied using radioisotope methodology.^80^ Here, the patient ingests radio-labeled water or food, the passage of which is measured by a gamma camera. Time-activity curves are recorded in the oropharyngeal and esophageal regions, and the stomach. Using a combination of electromyography (EMG) and scintigraphy, abnormal findings were detected in all of 18 PD patients, of whom only 13 complained of subjective dysphagia. Of note, delayed esophagus transit time was the most prevalent finding in the PD group.^81^

Other studies used simpler “glass of water” tests, where subjects are timed while drinking 150 mL of water. Here, 84% of PD patients fell >1SD below and 32% of patients fell >2SD below reference mean concerning swallowing rate (mL/s). Test performance correlated moderately with disease duration, but poorly with subjective symptoms of dysphagia.^82^ In a more detailed, quantitative water swallowing test, 58% of the H&Y stage 1 patients and >90% of H&Y stage 2–4 patients had abnormal results.^83^

To summarize, dysphagia is a frequent non-motor feature in PD, but often displays limited correlation to objective findings. Functional imaging studies show that most PD cases exhibit quantifiable dysmotility in the oro-pharynx and esophagus.^69^ Importantly, esophageal dysmotility seems to be even more prevalent than oropharyngeal dysfunction, which was further supported by the recent finding that 62 of 65 PD patients showed peristalsis dysfunction in the esophageal body using esophageal high-resolution manometry.^84^ The high prevalence of esophageal dysfunction may be caused by known aspects of PD pathology. The ganglia of the distal esophageal smooth muscle receive direct innervation from the DMV neurons, which are severely damaged in PD.^46^ Also, experimental lesions of the DMV markedly impairs motility patterns in the distal esophagus.^73^ Moreover, studies have documented that the esophageal mucosa exhibits the most extreme *α*-synuclein pathology throughout the gastrointestinal canal.^2, 3^ The high prevalence of subjective and objective gastroesophageal reflux in PD may similarly be explained by these pathologies, since the reflexive relaxation and constriction of the lower esophageal sphincter has a prominent vagovagal component.^85^

### Stomach

The stomach’s rhythmic contraction pattern are triggered by the interstitial cells of Cajal.^86^ However, considerable modulatory input from the autonomic and CNS is also involved in the control of volume, contraction strength, and acid secretion.^87, 88^

Bloating and abdominal fullness are seen in up to 50% of PD patients,^89^ and nausea and vomiting in 15% of cases.^90^ Whether gastroparesis is the underlying cause of these symptoms in PD is not fully determined. However, it seems probable that the presence of *α*-synuclein inclusions in enteric neurons and in vagal efferents to the stomach wall^2, 91^ is involved in perturbed gastric motility. It was recently shown that *α*-synuclein transgenic mice developed inclusion pathology in myenteric ganglia in a pattern closely mimicking the relative distribution of vagal efferents, and these mice developed gastroparesis and constipation.^92^

**Fig. 3:**
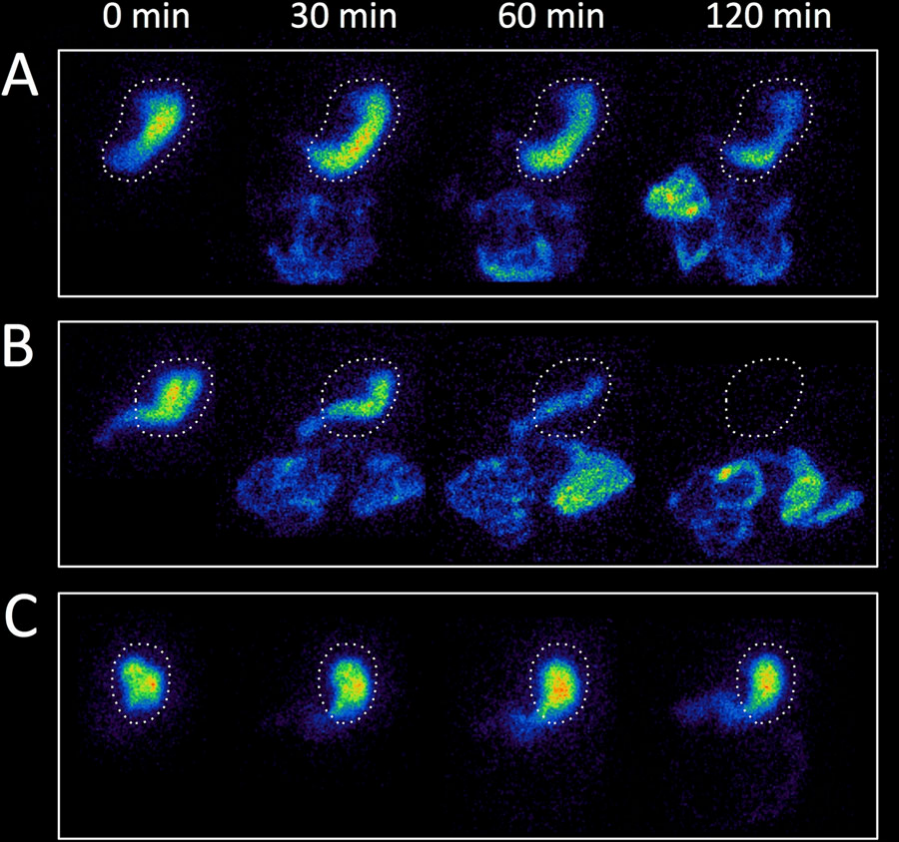
Gastric emptying scintigraphy images at 0, 30, 60, and 120 min after radioactive meal ingestion. **a** Healthy control with normal gastric emptying time (*T*_1/2_ 72 min). The *dashed line* designates the stomach. **b** PD patient with rapid gastric emptying time (*T*_1/2_ 26 min). **c** Vagotomized patient with severely increased gastric emptying time (*T*_1/2_ > 180 min)

The solid meal scintigraphical study is considered the gold standard for quantifying gastric emptying time (GET).^93^ After ingestion of a standard meal the radioactivity is recorded by serial images until gastric emptying is nearly complete. Figure 3 shows representative studies of subjects with normal, rapid, and severely delayed GET.

The presence of gastroparesis is often mentioned in the PD literature, but only few studies used gold standard solid meal scintigraphy to quantify GET in PD patients. One early paper reported no difference in GET between PD patients and matched controls.^94^ Another study reported that PD patients with pronounced on–off symptoms displayed prolonged GET in comparison to non-fluctuating patients, and both patient groups showed increased GET compared to the control group.^95^ Significantly delayed GET has been reported in familial PD, but in the same study idiopathic PD cases did not show significantly delayed GET.^96^ A recent study of 12 early stage PD patients reported significantly faster GET in the PD group compared to controls.^65^ In a comparison between treated and untreated PD cases, no significant difference in emptying time was seen.^97^ Finally, a recent study used liquid meal scintigraphy, and only three of 21 early PD patients showed prolonged GET.^98^

Thus, the majority of studies employing gold standard scintigraphic methodology do not support that prolonged GET is a frequent problem in PD, at least not at earlier disease stages. It is also not resolved if the presence of delayed GET shows robust correlations with subjective gastroparesis. The majority of studies reported wide ranges in GET among PD patients. Indeed, in a recent patient series two PD patients had very rapid GET (*T*_1/2_ < 30 min) indicative of a gastric dumping syndrome (Fig. 3b).^65^ Similar findings were also reported by other authors.^96, 97^

Although not an imaging modality, GET can also be measured with ^13^C-sodium breath tests. Here, a solid or liquid ^13^C-sodium octanoate-containing meal is ingested, absorbed in the proximal small intestine and converted by the liver to ^13^CO_2_. Subsequently, the expired ^13^CO_2_ concentration is quantified and mathematically transformed to an estimate of GET.^99^ The GET in PD patients has been studied using both solid and liquid meal breath tests, and most studies reported significantly prolonged GET in PD patients compared to control subjects.^99–105^ However, Goetze et al.^100^ found only significant difference between PD and HC when using solid meal but not with liquid meal. Recently, Epprecht et al.^101^ found no difference between early stage PD patients in the off state and controls. One study of RBD patients also found no difference compared to control subjects, suggesting that prolonged GET may not be a significant prodromal feature.^103^

Thus, discrepant findings are seen in the studies employing gold standard scintigraphy vs. breath test studies and this begs the question whether ^13^C-sodium breath tests are representative of gastric emptying in PD. The breath test depends on the combination of mechanic gastric emptying, adequate small intestine absorption and liver metabolism. The latter aspects have received little attention in the context of PD. Two studies reported pathological differential sugar absorption findings in PD, i.e., the amount of recovered mannitol was decreased compared to recovered lactulose in PD, suggesting a reduction in the absorptive intestinal surface in PD.^106, 107^Another study demonstrated increased intestinal permeability (gut leakiness) in PD.^108^ Nevertheless, the more consistently delayed GET in ^13^C-sodium studies may be explained by the combined pathologies of prolonged mechanical gastric emptying and small intestine malabsorption.

Finally, functional magnetic resonance imaging (MRI) of the stomach was able to detect a significant reduction in the amplitude of peristaltic contractions in PD patients, but the utility of this measure remains to be determined.^109^

### Small intestine

Very little is known about small intestinal function in PD. A recent Polish study compared the small bowel transit time in ten PD patients without gastrointestinal symptoms to ten matched controls. All subjects ingested a capsule containing the gamma emitting isotope 99m-technetium, which was followed using serial SPECT imaging.^110^ All healthy controls had anoro-cecal transit time <4 h, whereas seven PD patients had a transit time of >4 h (>24 h in one patient). A small pilot trial studied colon transit time in six PD cases using computed tomography (CT) scans and the radio-opaque marker method and in two of these patients radio-markers were still present in the small bowel, which is strongly suggestive of prolonged small intestinal transit time.^111^

An early case study reported severely dilated small intestine in a single PD patient.^112^ Interestingly, the small intestine of this patient contained large pockets of air, which could have been caused by small intestine anaerobic bacterial overgrowth now known to be highly prevalent in PD.^113^ However, it is unknown whether small intestine dilatation or presence of excessive amounts of air is a common finding in PD patients.

### Colon

Constipation is among the first non-motor symptoms to emerge in the prodromal phase of PD, appearing more than a decade before diagnosis in a sizeable fraction of PD patients.^7, 8, 114^ The overall prevalence of constipation in PD is approximately 40–50%, but with large variation across individual studies,^115–117^ probably resulting from the highly variable definitions of constipation used. Indeed, more than ten different definitions of constipation have been used in recent literature.^118^ Interestingly, the constipation prevalence in idiopathic RBD patients may be higher compared to PD patients without RBD.^119^

Widespread Lewy pathology is present in the nerve terminals of the submucosal and myenteric colonic plexus.^51, 52^ The parasympathetic innervation to the upper colon is supplied by the vagus, whereas the lower third is innervated by the sacral segment of the intermediolateral column. This parasympathetic input is intricately involved in the control of colon motility.^120, 121^ The parasympathetic neurons of the intermediolateral cell column also show Lewy pathology and cell degeneration in PD.^54^ Onuf’s nucleus and the lateral collateral region nucleus of the sacral spinal cord innervates the external anal and urethral sphincters^122^ and also show consistent heavy Lewy pathology in diagnosed PD cases.^54, 123^ The exact contribution of these multiple pathologies to colonic symptoms and perturbed motility in PD remains to be elucidated.

**Fig. 4:**
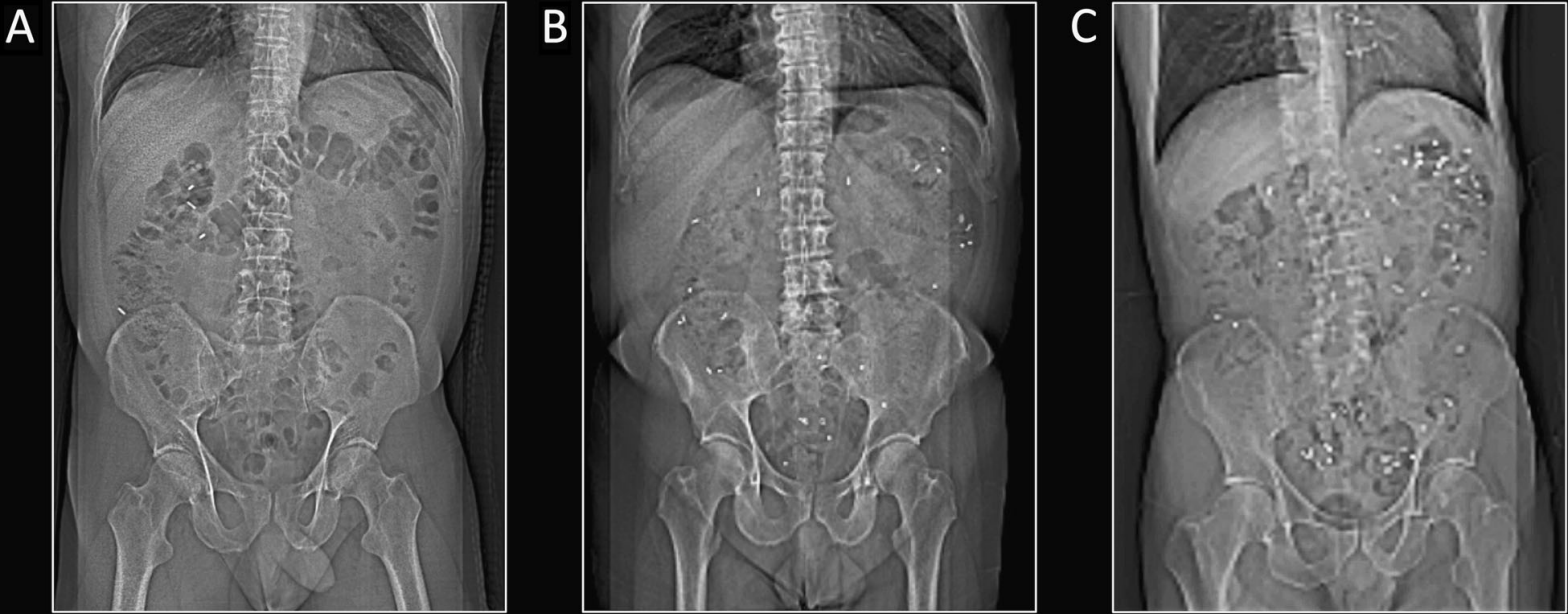
Radio-opaque marker studies of colonic transit time (*CTT*) using the 7-day (60 marker) protocol. **a** Healthy control with rapid CTT (4 markers). **b** PD patient with increased CTT (28 markers). **c** PD patient with severely increased CTT (57 markers). Note that the markers are situated mostly in the descending and recto-sigmoid colon suggestive of “outlet obstruction” constipation

Colonic transit time (CTT) can be measured using radio-opaque markers (ROM). Several radio-opaque plastic markers are ingested (one per day), and an abdominal X-ray image is acquired 24 h after the last capsule has been ingested. The CTT can then be estimated based on the number of retained ROM (Fig. 4).^124^ Several studies reported prolonged CTT in PD patients in comparison to matched control subjects.^125–128^ An early report suggested that the majority of newly diagnosed PD patients do not display prolonged CTT, but the authors applied very stringent criteria for defining prolonged CTT in the study.^129^ When applying the commonly accepted cutoff score for number of retained ROM,^124^ it is evident that 80% of newly diagnosed PD patients actually display increased CTT. One study detected no correlation between CTT and frequency of defecations per week or subjective constipation symptoms in PD patients. This suggests that objective CTT does not correlate with subjective constipation.^130^ Another study reported no difference in CTT between PD groups on and off levodopa treatment, which suggests colonicdys-motility is primarily caused by pathological processes.^131^

A uniform colonic distribution of markers is suggestive of slow-transit constipation, whereas an accumulation of markers in the distal colon is indicative of outlet-obstruction constipation (Fig. 4c).^132^ This distinction is of major importance in PD, since a substantial number of patients are unable to relax pelvic muscles during defecation (dyssynergia).^128, 133^ Indeed, straining for defecation is one of the most prevalent non-motor symptoms, underscoring that outlet-obstruction is of major significance in PD.^115, 134^

Three studies investigated the segmental distribution of colonic markers in PD patients. One study reported significantly increased rectosigmoid and total CTT in PD patients, whereas the difference in the right and left colon was not significant.^128^ Other studies showed noticeable long recto-sigmoid transit times compared to the other colonic segments, although they did not include control groups for comparison.^125, 131^ These observations suggest that prolonged CTT in PD is often of the “outlet obstruction” type. Furthermore, Wang et al.^125^ showed that subjective symptoms of bloating, manual evacuation of faeces, or regular use of suppositories was much more associated with prolonged CTT and anorectal dysfunction, than was frequency of bowel movements.

We assessed colon volume in 24 early stage PD patients and 15 controls using CT scans, and found significantly increased transverse and descending colon volume in the PD group.^111^ Such volumetric measures may provide new insights into gastrointestinal pathology in PD, and can be performed in a semi-automated fashion without radiation exposure using recently published MRI techniques.^135^ Also, a few early case series demonstrated mega-colon in late stage PD patients.^112, 136^ Severe dilation was seen throughout the colon but was most pronounced in the sigmoideum and rectum, which supports that constipation is often of the outlet obstruction type.

### Recto-anal imaging

Reflexive propulsion in the distal colon and rectum is under the control of lumbosacral defecation centers.^137, 138^ As mentioned above, sacral parasympathetic nuclei, including the nucleus of Onuf, exhibit consistent pathological *α*-synuclein inclusions in PD, which probably contributes to defecation dyssynergia. Pathology in more rostral centers in the CNS could, however, also be involved.

Rectoanal dysfunction is frequent in PD and is an important contributor to the high rate of constipation. Straining for defecation is the most prevalent gastrointestinal symptom in PD and is present in up to 83% of patients.^115, 134, 139^ One study showed that 67% of early PD patients display defecatory problems including straining and feelings of incomplete emptying. Importantly, only one third of these patients reported <3 bowel movements per week, which is a commonly used definition of constipation.^140^ Such symptoms of excessive straining and incomplete emptying are indicators of outlet obstruction constipation.^132^

Barium contrast defecography, in which a contrast agent is instilled rectally, is a standard method to assess rectoanal function. This technique provides measures of emptying rate and relaxation of pelvic floor muscles during defecation.^127, 132^ Simultaneous rectoanal manometry and EMG is often performed to measure the pressure phases during defecation and to monitor contractile function of the puborectalis muscle.^132^

An early study investigated rectoanal function in six PD cases, who were off medication, and showed paradox contractions of the sphincter and puborectalis muscles during straining and also incomplete emptying, with improvement subsequent to apomorphine.^133^ Other studies reported non-significantly increased rectal volume, significantly increased residual volumes after defecation, and decreased rectal contraction in PD ^128, 141^ However, Edwards et al.^127^ found no significant increase in post-defecation residuals in mild-to-moderate stage PD cases in the off state. A small study showed that botulinum toxin injection in the puborectalis muscle was efficacious in decreasing tonus in the anorectal muscle and an improvement in the anorectal angle was also seen during straining. Symptom improvement was also seen in 10 of 16 patients after a period of 2 months.^142^

## Urodynamic studies

Imaging of the urinary bladder is rarely considered an independent imaging modality, but rather one among several parameters in a complete urodynamic evaluation, which also includes EMG and pressure-flow measurements during bladder storage and micturition. For this reason, urodynamic imaging will be only briefly summarized here.

Bladder function and micturition involves numerous cortical and subcortical structures, including prefrontal and insular cortex, thalamus, basal ganglia, amygdala, and the periaqueductal grey,^143, 144^ and it has been hypothesized that bladder dysfunction in PD mainly stems from di- and tele-encephalic pathologies.^144, 145^ However, sympathetic and parasympathetic preganglionic cells in the intermediolateral cell columns of the lumbar-sacral medulla, Onuf’s nucleus, the raphe nuclei, and locus coeruleus are also intimately involved in bladder function,^122, 143^ and these structures are all predilection sites for marked and early neuronal damage in PD.^54, 123^ Thus, the pathophysiological substrate of urinary dysfunction may very likely be multi-focal. Interestingly, peripheral sympathetic denervation as measured by ^123^I-MIBG heart scintigraphy correlated with increasing severity of urinary symptoms, but not with any other non-motor symptom in PD.^146^

The prevalence of lower urinary tract symptoms ranges from 27% to 85% in diagnosed PD patients, and may also be increased in idiopathic RBD.^144, 147^ Nocturia, frequency, and urgency are the most prevalent bladder symptoms in PD. These symptoms are generally suggestive of detrusor overactivity, which has been corroborated in several urodynamic studies demonstrating detrusor over activity in 40–80% of medicated PD patients,^148–150^ and also in 58% of de novo untreated patients.^151^ The bladder volume at first desire to void is reduced in PD,^151, 152^ but detrusor activity during voiding is actually decreased in 50% of patients.^149^

## Imaging skin temperature

**Fig. 5:**
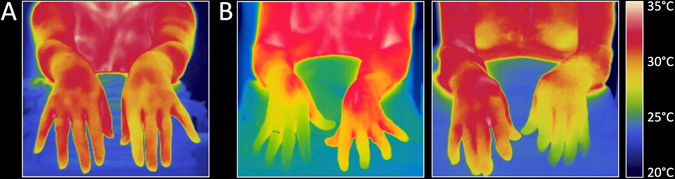
Thermograms in a healthy control subject (**a**) and two patients with PD (**b**). Note the asymmetric hand temperature in the patients. [Figure provided at the courtesy of the Dr Antonio-Rubio and co-authors]

The skin in PD patients shows autonomic denervation and *α*-synuclein pathology can be detected in cutaneous nerve terminals in most patients.^153, 154^ Thermal imaging was recently applied to study autonomic regulation of skin temperature in PD patients for the first time.^155^ The authors reported baseline asymmetry in hand temperature of most PD patients compared with controls (Fig. 5). During a cold stress test, in which one hand was immersed in cold water for 2 min, the non-immersed hand did not show a normal cooling pattern or thermal overshoot after immersion. After cooling the immersed hand displayed slower thermal recovery than seen in controls. These abnormal responses differentiated PD patients from controls with 87% sensitivity and 75–80% specificity.

## Conclusions

Non-invasive imaging tools present a unique opportunity to address the involvement of peripheral organs in PD pathophysiology. The majority of patients display a profound loss of cardiac sympathetic innervation. PET imaging with ^11^C-donepezil shows progressive signal loss in the intestine and pancreas in early-to-moderate stage PD, which may be a marker of parasympathetic denervation. Various functional imaging measures provide evidence for marked dysfunction throughout the gastrointestinal system. Importantly, objective dysfunction is often considerably more frequent than corresponding subjective symptoms. This mismatch between symptoms and objective markers is important, since subjective non-motor symptoms is currently utilized to define separate PD phenotypes,^156^ and to diagnose prodromal patients in the population.^157^ In summary, imaging measures of peripheral dysfunction are powerful tools for improving our knowledge of PD pathophysiology, guiding treatment, and for enhancing the accuracy of prodromal diagnosis.
